# Supplementary material for: Dynalign II: common secondary structure prediction for RNA homologs with domain insertions
Source: Nucleic Acids Res. 2014 Nov 21;42(22):13939–48. doi: 10.1093/nar/gku1172 (PMC4267632; doi:10.1093/nar/gku1172)
Supplement: SUPPLEMENTARY DATA [file supp_gku1172_nar-02021-z-2014-File012.zip › manual/javadoc/RNAstructure_java_drawing/source/dialogs/ImageDialog.html]

ImageDialog


JavaScript is disabled on your browser.


- Overview
- Package
- Class
- Tree
- Index
- Help

- Prev Class
- Next Class

- Frames
- No Frames

- All Classes

- Summary:
- Nested |
- Field |
- Constr |
- Method

- Detail:
- Field |
- Constr |
- Method


RNAstructure\_java\_drawing.source.dialogs

## Class ImageDialog

- java.lang.Object
- - java.awt.Component
  - - java.awt.Container
    - - java.awt.Window
      - - java.awt.Dialog
        - - javax.swing.JDialog
          - - RNAstructure\_java\_drawing.source.dialogs.ImageDialog

- All Implemented Interfaces:
  :   ImageObserver, MenuContainer, Printable, Serializable, Accessible, RootPaneContainer, WindowConstants

  Direct Known Subclasses:
  :   PlotDialog, StructureDialog

  ---

    

  ```
  public abstract class ImageDialog
  extends JDialog
  implements Printable
  ```

  A printable and zoomable dialog that holds drawn images.

  See Also:
  :   Serialized Form

- - ### Nested Class Summary

    Nested Classes

    | Modifier and Type | Class and Description |
    | --- | --- |
    | `private class` | `ImageDialog.PrintMenu` An inner class which creates a print menu. |
    | `private class` | `ImageDialog.ZoomDialog` An inner class which creates a dialog that zooms a structure. |

    - ### Nested classes/interfaces inherited from class javax.swing.JDialog

      `JDialog.AccessibleJDialog`
    - ### Nested classes/interfaces inherited from class java.awt.Dialog

      `Dialog.AccessibleAWTDialog, Dialog.ModalExclusionType, Dialog.ModalityType`
    - ### Nested classes/interfaces inherited from class java.awt.Window

      `Window.AccessibleAWTWindow, Window.Type`
    - ### Nested classes/interfaces inherited from class java.awt.Container

      `Container.AccessibleAWTContainer`
    - ### Nested classes/interfaces inherited from class java.awt.Component

      `Component.AccessibleAWTComponent, Component.BaselineResizeBehavior, Component.BltBufferStrategy, Component.FlipBufferStrategy`
  - ### Field Summary

    Fields

    | Modifier and Type | Field and Description |
    | --- | --- |
    | `protected String` | `error` The error message generated while building this panel, if any. |
    | `protected String` | `file` The file that is drawn in some way on this panel. |
    | `private PrinterJob` | `job` The job that prints out this panel |
    | `private JLabel` | `label` The main label that describes the panel. |
    | `private JPanel` | `legendGrid` The panel that contains the legend grid. |
    | `protected JPanel` | `mainPanel` The main panel that the drawing resides on. |
    | `protected double` | `maxBoundX` The maximum X bound of the image. |
    | `protected double` | `maxBoundY` The maximum Y bound of the image. |
    | `protected int` | `preferredDialogWidth` The preferred dialog width. |
    | `protected double` | `scale` The scale at which the image is seen. |
    | `private static long` | `serialVersionUID` |

    - ### Fields inherited from class javax.swing.JDialog

      `accessibleContext, rootPane, rootPaneCheckingEnabled`
    - ### Fields inherited from class java.awt.Dialog

      `DEFAULT_MODALITY_TYPE`
    - ### Fields inherited from class java.awt.Component

      `BOTTOM_ALIGNMENT, CENTER_ALIGNMENT, LEFT_ALIGNMENT, RIGHT_ALIGNMENT, TOP_ALIGNMENT`
    - ### Fields inherited from interface java.awt.print.Printable

      `NO_SUCH_PAGE, PAGE_EXISTS`
    - ### Fields inherited from interface javax.swing.WindowConstants

      `DISPOSE_ON_CLOSE, DO_NOTHING_ON_CLOSE, EXIT_ON_CLOSE, HIDE_ON_CLOSE`
    - ### Fields inherited from interface java.awt.image.ImageObserver

      `ABORT, ALLBITS, ERROR, FRAMEBITS, HEIGHT, PROPERTIES, SOMEBITS, WIDTH`
  - ### Constructor Summary

    Constructors

    | Modifier | Constructor and Description |
    | --- | --- |
    | `protected` | `ImageDialog(String file)` Constructor. |
  - ### Method Summary

    Methods

    | Modifier and Type | Method and Description |
    | --- | --- |
    | `void` | `buildLegend(String... data)` Build the legend grid for this panel. |
    | `protected void` | `createDefaultMenuBar(RNAstructureMenu... menus)` Create the image dialog's default menu bar. |
    | `protected abstract void` | `createDrawnImage(Graphics2D g2)` Create the image drawn on this panel. |
    | `String` | `getFile()` Get the file drawn on this dialog. |
    | `double` | `getScale()` Get the scale at which the image is zoomed. |
    | `boolean` | `isError()` Get whether this panel ran into an error while being built. |
    | `int` | `print(Graphics graphics, PageFormat format, int index)` Render the panel for the print job. |
    | `void` | `printPanel()` Print if the user decides to, then reset the printer job properties. |
    | `protected abstract void` | `readImageData()` Read image data from a particular file. |
    | `void` | `refresh()` Refresh this dialog. |
    | `void` | `setScale(int scale)` Set the image scale. |
    | `void` | `setTopCaption(String text)` Set the text on the info label. |
    | `void` | `viewDialog()` View the dialog. |
    | `void` | `viewZoomDialog()` View a zooming dialog. |
    | `void` | `zoomImage()` Zoom the image on the panel. |

    - ### Methods inherited from class javax.swing.JDialog

      `addImpl, createRootPane, dialogInit, getAccessibleContext, getContentPane, getDefaultCloseOperation, getGlassPane, getGraphics, getJMenuBar, getLayeredPane, getRootPane, getTransferHandler, isDefaultLookAndFeelDecorated, isRootPaneCheckingEnabled, paramString, processWindowEvent, remove, repaint, setContentPane, setDefaultCloseOperation, setDefaultLookAndFeelDecorated, setGlassPane, setJMenuBar, setLayeredPane, setLayout, setRootPane, setRootPaneCheckingEnabled, setTransferHandler, update`
    - ### Methods inherited from class java.awt.Dialog

      `addNotify, getModalityType, getTitle, hide, isModal, isResizable, isUndecorated, setBackground, setModal, setModalityType, setOpacity, setResizable, setShape, setTitle, setUndecorated, setVisible, show, toBack`
    - ### Methods inherited from class java.awt.Window

      `addPropertyChangeListener, addPropertyChangeListener, addWindowFocusListener, addWindowListener, addWindowStateListener, applyResourceBundle, applyResourceBundle, createBufferStrategy, createBufferStrategy, dispose, getBackground, getBufferStrategy, getFocusableWindowState, getFocusCycleRootAncestor, getFocusOwner, getFocusTraversalKeys, getIconImages, getInputContext, getListeners, getLocale, getModalExclusionType, getMostRecentFocusOwner, getOpacity, getOwnedWindows, getOwner, getOwnerlessWindows, getShape, getToolkit, getType, getWarningString, getWindowFocusListeners, getWindowListeners, getWindows, getWindowStateListeners, isActive, isAlwaysOnTop, isAlwaysOnTopSupported, isAutoRequestFocus, isFocusableWindow, isFocusCycleRoot, isFocused, isLocationByPlatform, isOpaque, isShowing, isValidateRoot, pack, paint, postEvent, processEvent, processWindowFocusEvent, processWindowStateEvent, removeNotify, removeWindowFocusListener, removeWindowListener, removeWindowStateListener, reshape, setAlwaysOnTop, setAutoRequestFocus, setBounds, setBounds, setCursor, setFocusableWindowState, setFocusCycleRoot, setIconImage, setIconImages, setLocation, setLocation, setLocationByPlatform, setLocationRelativeTo, setMinimumSize, setModalExclusionType, setSize, setSize, setType, toFront`
    - ### Methods inherited from class java.awt.Container

      `add, add, add, add, add, addContainerListener, applyComponentOrientation, areFocusTraversalKeysSet, countComponents, deliverEvent, doLayout, findComponentAt, findComponentAt, getAlignmentX, getAlignmentY, getComponent, getComponentAt, getComponentAt, getComponentCount, getComponents, getComponentZOrder, getContainerListeners, getFocusTraversalPolicy, getInsets, getLayout, getMaximumSize, getMinimumSize, getMousePosition, getPreferredSize, insets, invalidate, isAncestorOf, isFocusCycleRoot, isFocusTraversalPolicyProvider, isFocusTraversalPolicySet, layout, list, list, locate, minimumSize, paintComponents, preferredSize, print, printComponents, processContainerEvent, remove, removeAll, removeContainerListener, setComponentZOrder, setFocusTraversalKeys, setFocusTraversalPolicy, setFocusTraversalPolicyProvider, setFont, transferFocusDownCycle, validate, validateTree`
    - ### Methods inherited from class java.awt.Component

      `action, add, addComponentListener, addFocusListener, addHierarchyBoundsListener, addHierarchyListener, addInputMethodListener, addKeyListener, addMouseListener, addMouseMotionListener, addMouseWheelListener, bounds, checkImage, checkImage, coalesceEvents, contains, contains, createImage, createImage, createVolatileImage, createVolatileImage, disable, disableEvents, dispatchEvent, enable, enable, enableEvents, enableInputMethods, firePropertyChange, firePropertyChange, firePropertyChange, firePropertyChange, firePropertyChange, firePropertyChange, firePropertyChange, firePropertyChange, firePropertyChange, getBaseline, getBaselineResizeBehavior, getBounds, getBounds, getColorModel, getComponentListeners, getComponentOrientation, getCursor, getDropTarget, getFocusListeners, getFocusTraversalKeysEnabled, getFont, getFontMetrics, getForeground, getGraphicsConfiguration, getHeight, getHierarchyBoundsListeners, getHierarchyListeners, getIgnoreRepaint, getInputMethodListeners, getInputMethodRequests, getKeyListeners, getLocation, getLocation, getLocationOnScreen, getMouseListeners, getMouseMotionListeners, getMousePosition, getMouseWheelListeners, getName, getParent, getPeer, getPropertyChangeListeners, getPropertyChangeListeners, getSize, getSize, getTreeLock, getWidth, getX, getY, gotFocus, handleEvent, hasFocus, imageUpdate, inside, isBackgroundSet, isCursorSet, isDisplayable, isDoubleBuffered, isEnabled, isFocusable, isFocusOwner, isFocusTraversable, isFontSet, isForegroundSet, isLightweight, isMaximumSizeSet, isMinimumSizeSet, isPreferredSizeSet, isValid, isVisible, keyDown, keyUp, list, list, list, location, lostFocus, mouseDown, mouseDrag, mouseEnter, mouseExit, mouseMove, mouseUp, move, nextFocus, paintAll, prepareImage, prepareImage, printAll, processComponentEvent, processFocusEvent, processHierarchyBoundsEvent, processHierarchyEvent, processInputMethodEvent, processKeyEvent, processMouseEvent, processMouseMotionEvent, processMouseWheelEvent, remove, removeComponentListener, removeFocusListener, removeHierarchyBoundsListener, removeHierarchyListener, removeInputMethodListener, removeKeyListener, removeMouseListener, removeMouseMotionListener, removeMouseWheelListener, removePropertyChangeListener, removePropertyChangeListener, repaint, repaint, repaint, requestFocus, requestFocus, requestFocusInWindow, requestFocusInWindow, resize, resize, revalidate, setComponentOrientation, setDropTarget, setEnabled, setFocusable, setFocusTraversalKeysEnabled, setForeground, setIgnoreRepaint, setLocale, setMaximumSize, setName, setPreferredSize, show, size, toString, transferFocus, transferFocusBackward, transferFocusUpCycle`
    - ### Methods inherited from class java.lang.Object

      `clone, equals, finalize, getClass, hashCode, notify, notifyAll, wait, wait, wait`

- - ### Field Detail


    - #### error

      ```
      protected String error
      ```

      The error message generated while building this panel, if any.


    - #### file

      ```
      protected String file
      ```

      The file that is drawn in some way on this panel.


    - #### job

      ```
      private PrinterJob job
      ```

      The job that prints out this panel


    - #### label

      ```
      private JLabel label
      ```

      The main label that describes the panel.


    - #### legendGrid

      ```
      private JPanel legendGrid
      ```

      The panel that contains the legend grid.


    - #### mainPanel

      ```
      protected JPanel mainPanel
      ```

      The main panel that the drawing resides on.


    - #### maxBoundX

      ```
      protected double maxBoundX
      ```

      The maximum X bound of the image.


    - #### maxBoundY

      ```
      protected double maxBoundY
      ```

      The maximum Y bound of the image.


    - #### preferredDialogWidth

      ```
      protected final int preferredDialogWidth
      ```

      The preferred dialog width.

      See Also:
      :   Constant Field Values


    - #### scale

      ```
      protected double scale
      ```

      The scale at which the image is seen.


    - #### serialVersionUID

      ```
      private static final long serialVersionUID
      ```

      See Also:
      :   Constant Field Values
  - ### Constructor Detail


    - #### ImageDialog

      ```
      protected ImageDialog(String file)
      ```

      Constructor.

      Parameters:
      :   `file` - The file that is drawn in some way.
  - ### Method Detail


    - #### buildLegend

      ```
      public void buildLegend(String... data)
      ```

      Build the legend grid for this panel.

      Parameters:
      :   `data` - The array of data for the legend.


    - #### createDefaultMenuBar

      ```
      protected void createDefaultMenuBar(RNAstructureMenu... menus)
      ```

      Create the image dialog's default menu bar.

      Parameters:
      :   `menus` - The unique menus attacted to this dialog.


    - #### createDrawnImage

      ```
      protected abstract void createDrawnImage(Graphics2D g2)
      ```

      Create the image drawn on this panel.

      Parameters:
      :   `g2` - The graphics context.


    - #### getFile

      ```
      public String getFile()
      ```

      Get the file drawn on this dialog.

      Returns:
      :   The file name.


    - #### getScale

      ```
      public double getScale()
      ```

      Get the scale at which the image is zoomed.

      Returns:
      :   The scale.


    - #### isError

      ```
      public boolean isError()
      ```

      Get whether this panel ran into an error while being built.

      Returns:
      :   True if an error occurred, false if not.


    - #### print

      ```
      public int print(Graphics graphics,
              PageFormat format,
              int index)
      ```

      Render the panel for the print job.

      **Specified by:**
      :   `print` in interface `Printable`

      Parameters:
      :   `graphics` - The graphics object that is printed.
      :   `format` - The page format.
      :   `index` - The page index


    - #### printPanel

      ```
      public void printPanel()
      ```

      Print if the user decides to, then reset the printer job properties.


    - #### readImageData

      ```
      protected abstract void readImageData()
      ```

      Read image data from a particular file.


    - #### refresh

      ```
      public void refresh()
      ```

      Refresh this dialog.


    - #### setScale

      ```
      public void setScale(int scale)
      ```

      Set the image scale.

      Parameters:
      :   `scale` - The percent by which the image is scaled,
          as an integer (ex. 5 = 5%).


    - #### setTopCaption

      ```
      public void setTopCaption(String text)
      ```

      Set the text on the info label.

      Parameters:
      :   `text` - The text to set on the label.


    - #### viewDialog

      ```
      public void viewDialog()
      ```

      View the dialog.


    - #### viewZoomDialog

      ```
      public void viewZoomDialog()
      ```

      View a zooming dialog.


    - #### zoomImage

      ```
      public void zoomImage()
      ```

      Zoom the image on the panel.


- Overview
- Package
- Class
- Tree
- Index
- Help

- Prev Class
- Next Class

- Frames
- No Frames

- All Classes

- Summary:
- Nested |
- Field |
- Constr |
- Method

- Detail:
- Field |
- Constr |
- Method
